# Soluble ST2 as a Biomarker for Early Complications in Patients with Chronic Thromboembolic Pulmonary Hypertension Treated with Balloon Pulmonary Angioplasty

**DOI:** 10.3390/diagnostics11010133

**Published:** 2021-01-16

**Authors:** Marta Banaszkiewicz, Arkadiusz Pietrasik, Michał Florczyk, Piotr Kędzierski, Michał Piłka, Rafał Mańczak, Janusz Kochman, Grzegorz Opolski, Adam Torbicki, Marcin Kurzyna, Szymon Darocha

**Affiliations:** 1 Department of Pulmonary Circulation, Thromboembolic Diseases and Cardiology, Centre of Postgraduate Medical Education, European Health Center Otwock, 05-400 Otwock, Poland; marta.banaszkiewicz@gmail.com (M.B.); michal.florczyk@ecz-otwock.pl (M.F.); piotr.kedzierski@ecz-otwock.pl (P.K.); michal.pilka@ecz-otwock.pl (M.P.); rafal.manczak@ecz-otwock.pl (R.M.); adam.torbicki@ecz.otwock.pl (A.T.); marcin.kurzyna@ecz-otwock.pl (M.K.); szymon.darocha@ecz-otwock.pl (S.D.); 2 Department and Faculty of Cardiology, Medical University of Warsaw, 02-097 Warsaw, Poland; jkochman@wum.edu.pl (J.K.); grzegorz.opolski@wum.edu.pl (G.O.)

**Keywords:** chronic thromboembolic pulmonary hypertension, balloon pulmonary angioplasty, soluble ST2

## Abstract

Background: The aim of the study was to assess soluble ST2 (sST2) concentration and its dynamic changes in the periprocedural period in patients with chronic thromboembolic pulmonary hypertension (CTEPH) treated with balloon pulmonary angioplasty (BPA). Methods: We prospectively analyzed 57 procedures of BPA performed in 37 patients with CTEPH. Biomarkers, such as N-terminal pro B-type natriuretic peptide (NT-proBNP), troponin T (TnT), and sST2 were assessed at four time points: Before the BPA procedure, 24 h and 48 h after the procedure, and at the discharge from hospital. Each postprocedural period was assessed for complications. Results: Before the BPA procedure, median sST2 concentration was 26.56 ng/mL (IQR: 16.66–40.83 ng/mL). sST2 concentration was significantly higher 24 h and 48 h after the BPA compared to the baseline measurements (33.31 ng/mL (IQR: 20.81–62.56), *p* = 0.000 and 27.45 ng/mL (IQR: 17.66–54.45), *p* = 0.028, respectively). sST2 level 24 h after the BPA procedure was significantly higher in the group with complications compared to the group without complications in the postprocedural period (97.66 ng/mL (IQR: 53.07–126.18) vs. 26.86 ng/mL (IQR: 19.10–40.12), *p* = 0.000). Conclusions: sST2 concentration in patients with CTEPH treated with BPA changes significantly in the postprocedural period and is significantly higher in the group with complications in postprocedural period.

## 1. Introduction

Chronic thromboembolic pulmonary hypertension (CTEPH) is one of the types of pulmonary hypertension (PH) caused by persisting pulmonary arteries obstruction, which consequently leads to elevated pulmonary vascular resistance (PVR) and right ventricular (RV) overload. In the natural course of disease progression, the compensatory right heart adaptation gradually fails and progressive dysfunction of RV ensues, finally resulting in RV failure, which is associated with worse prognosis and outcomes [1,2]. The gold standard of treatment in CTEPH patients is pulmonary endarterectomy (PEA) [3]. However, for inoperable patients, in addition to targeted pharmacotherapy, balloon pulmonary angioplasty (BPA) may be considered as an interventional treatment option [4]. Due to improvement in the skills of BPA and optimization of periprocedural care, the rate of complications of BPA procedures has decreased over the years, but complications continue to occur and can be significant.

The soluble ST2 protein is an interleukin (IL)-1 receptor family member, that exists as transmembrane and soluble isoforms, which are both receptors for IL-33 [5,6,7]. sST2 is supposed to act as an opposing decoy receptor for IL-33, thus inhibiting the IL-33/ST2L signaling course. The soluble ST2 is expressed in various cell types (e.g., cardiomyocytes, endothelial cells, and smooth cells) [8,9], released predominantly in response to myocardial strain and remodeling [10], but is also linked to inflammation processes and tissue injury [11]. Besides troponins and natriuretic peptides, soluble ST2 protein could be an additional marker for adverse outcomes in patients with heart failure (HF) [6,12,13]. Previous studies revealed elevated sST2 concentrations not only in cardiac diseases, but also in pulmonary and inflammatory disorders [5,14,15]. sST2 has been already recommended for an additive risk stratification in the American Heart Association guidelines for the management of HF [16]. However, there are limited data on the use of sST2 in patients with right heart failure secondary to PH [14,17,18]. There are insufficient data on the clinical usefulness of sST2 biomarker measurements in patients with CTEPH, especially when the interventional treatment is used. This study assessed sST2 concentration and its changes in the periprocedural period in patients with CTEPH treated with balloon pulmonary angioplasty.

## 2. Materials and Methods

### 2.1. Study Population

This is a prospective, observational study without study sample calculation. The study was performed between July 2016 and June 2017 at the European Health Centre in the Department of Pulmonary Circulation, Thromboembolic Disease and Cardiology, Centre of Postgraduate Medical Education in Otwock, Poland, in association with the 1st Chair and Department of Cardiology, Medical University of Warsaw, Poland. The ethics board of the Medical University of Warsaw approved the study (No. KB/181/2016). All study participants gave written informed consent prior to inclusion. We prospectively analyzed the course of 57 consecutive BPA procedures performed in 37 patients diagnosed with CTEPH, including the postprocedural period. The CTEPH diagnosis, therapeutic decisions, and BPA therapy were performed in accordance with current guidelines [3]. All the patients were previously classified as ineligible for PEA and qualified for BPA by an interdisciplinary CTEPH team consisting of an interventional cardiologist with experience in BPA, a cardiac surgeon experienced in PEA and a cardiologist experienced in therapy for pulmonary hypertension. Additionally, all the patients received the targeted therapy with sildenafil or riociguat.

### 2.2. Baseline Tests and Measurements

In accordance with the schedule, all patients were admitted to the hospital a day before the BPA procedure was performed. On the admission day, WHO functional class (FC) and biochemical parameters, including N-terminal pro B-type natriuretic peptide (NT-proBNP) and troponin T (TnT) concentration, were assessed. Immediately before the BPA procedure, right heart catheterization (RHC) was performed pursuant to current guidelines [19].

### 2.3. Balloon Pulmonary Angioplasty, Periprocedural Period and Complications

The description of BPA procedures performed at our center has been previously published [20,21,22,23]. In brief, BPA was carried out via access through the right femoral vein. During the procedure, 2000 units per hour of unfractionated heparin was used. Chronic antithrombotic treatment with direct oral anticoagulants or vitamin K antagonists was discontinued 24 h before the procedure. Patients treated with long-term oxygen therapy, received oxygen at the same constant flow rate throughout the procedure. A 6-F coronary guiding catheter (Launcher; Medtronic, Minneapolis, MN, USA) was inserted into the pulmonary artery using a 90-cm 6-F vascular sheath (Flexor; Cook, Bloomington, IN, USA). The balloon catheter size was attuned to the type of lesion, degree of stenosis of the pulmonary artery determined by angiography, intravascular ultrasound examination (IVUS), and severity of PH [23]. The course of each postprocedural period was analyzed for complications. A postprocedural complications were defined as the occurrence of lung injury (LI) of grade 3 or higher according to the Inami classification [24], infections (increase in laboratory markers for inflammation with clinical signs of infection), and any other clinical conditions leading to exacerbation of HF (e.g., sustained arrythmia). For further analysis, the studied postprocedural courses were divided into two subgroups: Those without complications and those with complications.

### 2.4. sST2 Measurements

Biomarkers analysis, including NT-proBNP and TnT assessment and blood collection for the sST2 assessment, was performed in the study population at four time points: On admission to the hospital, 24 h after the BPA procedure, 48 h after the BPA procedure, and on discharge from the hospital. The blood samples for sST2 evaluation were obtained from the peripheral vein. After collection of EDTA anticoagulated blood samples, plasma aliquots were frozen at −80 °C until further analysis. All the sST2 concentration measurements were performed within 3 months from the date of samples collection. The sST2 concentration was measured using the ASPECT-PLUS sST2 test (Critical Diagnostics, San Diego, CA, USA). The ASPECT-PLUS sST2 test is a rapid lateral flow immunoassay for the quantitative measurement of sST2 in human plasma, using a specially adapted reader (the ASPECT Reader). The ASPECT-PLUS ST2 test cassette contains goat polyclonal antibodies against murine IgG, murine mouse monoclonal antibodies against human sST2, and a fluorescent dye. The ASPECT Reader measures the fluorescence signal and quantitative sST2 values are delivered as nanograms per milliliter. The plasma sST2 concentration was assessed in line with the manufacturer’s manual. Based on the measurement limits of the ASPECT Reader, an sST2 plasma concentration >250 ng/mL or <12.5 ng/mL was reported as being above or below the limit values, respectively. The analysis procedure meets the admissible criteria for analytical parameters, with an average coefficient of variation (CV) of 10.4% for the variation within the assay and 13.6% for the variation among assays.

### 2.5. Statistical Analysis

Normally distributed continuous variables were presented as mean values and standard deviations, whereas nonnormally distributed continuous variables were presented as median values with 25th and 75th percentile values, i.e., interquartile range (IQR). Nominal variables were presented as numbers and percentages. Independent subcohorts were compared using Mann–Whitney-U test for continuous variables and using Students-t test for normally distributed parameters. Dependent observations were compared with Wilcoxon’s paired test. Receiver operating characteristic curves were plotted for baseline sST2 concentration [25]. In addition, the Youden statistics was performed to determine the baseline biomarker (sST2) cut-off for prediction of complications in the postprocedural period. A *p* value ≤ 0.05 was considered significant for all tests. R software, version 3.4.3 (The R Foundation for Statistical Computing, 2016, Vienna, Austria) was used for all analysis.

## 3. Results

A total of 57 consecutive BPA procedures performed in 37 CTEPH patients were included in this study (21 females, mean age 52 ± 19 years). Of this number, 17 patients (46%) were treated with sildenafil, while 20 patients were treated with riociguat. Median length of hospitalization in all study population was five days (IQR: 5–7). The baseline characteristics of all studied population is summarized in Table 1.

Further measurements in the whole studied group revealed a significantly higher sST2 concentration 24 h and 48 h after the BPA procedure, compared to the admission day measurements (33.31 ng/mL (IQR: 20.81–62.56), *p* = 0.000 and 27.45 ng/mL (IQR: 17.66–54.45), *p* = 0.028, respectively). In the analysis of all the procedures included in the study, the sST2 concentration obtained on the day of discharge from the hospital was significantly lower compared to the baseline measurements (21.59 pg/mL (IQR: 12.50–33.48) vs. 26.56 pg/mL (IQR: 16.66–40.83), *p* = 0.048). The dynamics of NT-proBNP and TnT concentrations were different, the concentrations of these biomarkers decreased 24 and 48 h after the procedure, with the lowest concentration recorded on the day of hospital discharge. Overall sST2, NT-proBNP and TnT measurements in periprocedural period at each time point are presented in Table 2.

The analysis of the course of postprocedural period revealed 42 cases with no complications in the postprocedural periods and 15 cases with complications in the postprocedural periods. Among the cases with complications, we distinguished: 11 cases of LI classified as grade 3 in accordance with the classification proposed by Inami, two cases of LI requiring non-invasive ventilation—classified as grade 4 by Inami et al., one case of Tako—Tsubo cardiomyopathy, and one case of bilateral pneumonia. Median length of hospitalization in the group with no postprocedural complications was five days [IQR: 5–5] whereas it was seven days (IQR: 6–9) in the group with complications. There were significant differences between the group with no complications and the group with complications in the postprocedural period in mean pulmonary artery pressure(mPAP) (41.6 ± 10.1 vs. 50.3 ± 10.07 mmHg, *p* = 0.006), pulmonary vascular resistance (PVR) (7.0 ± 3.6 vs. 9.5 ± 4.1 WU, *p* = 0.025), mixed venous oxygen saturation (MVsatO2) (69.1 ± 5.4 vs. 63.7 ± 7.1 %, *p* = 0.009), and aortic oxygen saturation (AOsatO2)(96.3 ± 2.6 vs. 93.7 ± 2.9%, *p* = 0.005. Hemodynamic parameters for groups with and without complications in the postprocedural period are presented in Table 3.

**Figure 1:**
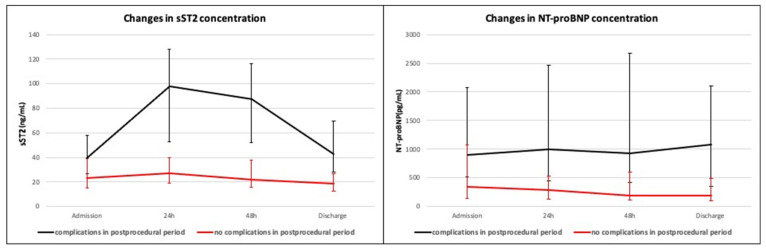
Changes in sST2 and NT-proBNP concentration in the groups with and without complications in the postprocedural period.

Further analysis revealed significant differences in sST2 concentration between cases with and without complications in the postprocedural period. The median sST2 concentration was significantly higher in group with complications compared to group without complications in the postprocedural period at each measurement time point. Significant differences were also observed between subgroups for the NT-proBNP concentrations. In the analysis of TnT concentrations at four measurement time points, no significant differences between two subgroups were observed. Table 4 presents concentrations of sST2, NT-proBNP and TnT separately for groups with and without complications in the postprocedural period at four time points. Figure 1 presents the curves of change in sST2 and NT-proBNP concentrations for the groups with and without complications in the postprocedural period.

**Figure 2:**
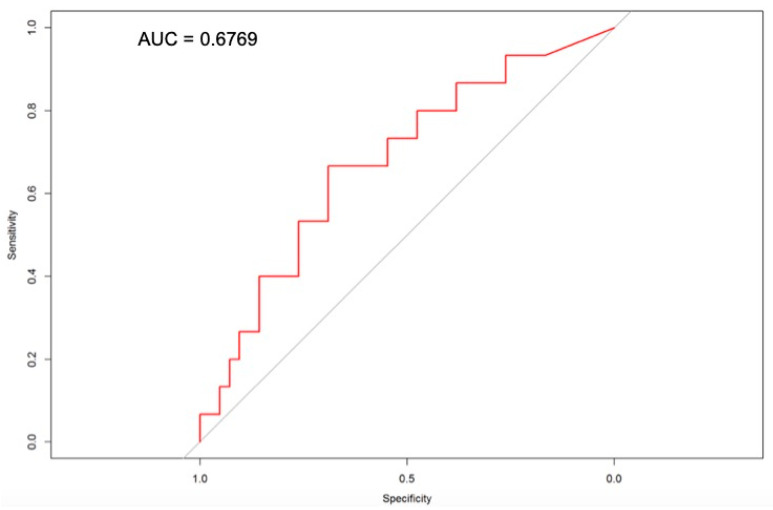
Receiver operating curve for sST2 concentration assessed on admission day in relation to the concentration during the postprocedural period with complications. AUC—area under the curve.

ROC analysis revealed that the area under the curve (AUC) for sST2 concentration assessed at hospital admission for prediction of complications in the postprocedural period was 0.6769. sST2 concentration assessed at admission day of 31.48 ng/mL or higher had a sensitivity of 66% and specificity of 69% for prediction of complications in postprocedural period (Figure 2).

## 4. Discussion

Soluble ST2 is the marker for inflammatory tissue damage [8,26], cardiac mechanical strain, and fibrosis [10,11], all of which may be engaged in the development of heart failure in patients diagnosed with CTEPH. In the last couple of years, numerous researchers have tried to assess reference values for sST2 in healthy populations, exposing wide range and noteworthy overlap between healthy individuals and people with heart failure. Results obtained by Lu et al. revealed that in healthy volunteers the interquartile values of sST2 concentration were between 15 and 25 ng/mL, whereas sST2 level of 35 ng/mL falls between 90th and 95th centiles of healthy population [27]. Moreover, in patients with heart failure, sST2 level above 35 ng/mL was linked to a higher risk of adverse events (defined as hospitalization or death in one year), compared to patients with sST2 levels below this value [28,29,30]. In patients with right heart failure due to PH, elevated sST2 concentrations were associated with right ventricular remodeling [14] and correlated both with biochemical parameters such as NT-proBNP or TnT and hemodynamic parameters [17,18,31].

The best of our knowledge, this is the very first study to assess soluble ST2 concentration in early postprocedural period in CTEPH patients treated with BPA. Previous studies have evaluated numerous biomarkers in cohorts of patients with CTEPH or other types of PH, but a specific biomarker especially useful for monitoring early complications and outcomes of interventional treatment is still continuously sought.

Pathological changes of pulmonary hemodynamics in patients with CTEPH generate the impairment of right ventricle function, resulting in increased right ventricular afterload, increased wall tension, and myocardial damage [32,33]. BPA is a promising treatment method for patients with inoperable CTEPH [4]. In the last few years, the data about the beneficial effects and technical improvements of the BPA strategy led to a high level of peri- and postprocedural safety [20,21,22,23,34]. Despite this, the assessment of biomarkers for heart failure, not only in monitoring treatment effects but also the periprocedural course and possible postprocedural complications, seems to be an important point in the entire therapeutic process of these patients.

The main findings of this study are: (i) Serum sST2 concentration is increased in CTEPH patients, with significantly higher baseline sST2 concentration in patients with complications in postprocedural period; (ii) serum sST2 levels significantly increased early after the BPA procedure, regardless of complications in the postprocedural period; (iii) on the contrary, no similar increase in NT-proBNP concentration was observed in the group with no complications, which may suggest a predominant non-cardiac source of sST2 in CTEPH patients.

sST2 levels obtained in our study in all population on admission are slightly higher than levels found in healthy population assessed in previous studies (26.56 ng/mL vs. 25 ng/mL). However, it is worth noting that the baseline concentration of sST2 in the group with postprocedural complications is similar to the cutoff value for sST2 associated with an unfavorable prognosis for patients with heart failure and higher than median sST2 levels obtained in CTEPH population in a study performed by Geenen et al. (39.73 ng/mL vs. 34.8 ng/mL) [17]. This may suggest that a higher baseline sST2 concentration is associated with a higher risk of complications in the postprocedural period.

Furthermore, the ROC analysis in our study revealed the AUC 0.6769 for sST2 level assessed on admission for predicting postprocedural complications. The cut-off for sST2 level assessed on admission associated with higher risk of complications in postprocedural period was 31.48 ng/mL. This value is lower compared to reports from previous studies. Placido at el. reported increased risk of mortality for patients diagnosed with PH who have sST2 level above 68.6 ng/mL [35]. It is worth emphasizing that in our study we only analyzed early postprocedural complications and not long-term prognosis of patients with PH as conducted by Placido et al.

Rather than relying on a single measurement for prognosis in patients with HF, emerging data suggest a potential in using serial levels of sST2 in heart failure management, assessment of treatment effects, and monitoring of disease severity. In a study conducted by Manzano-Fernandez et al. on 72 patients with acute decompensated HF, sST2 values >76 ng/mL at presentation and >46 ng/mL at day 4 following hospitalization identified those with a 50% risk of mortality, transplantation, or rehospitalization [36]. In patients with chronic HF, sST2 levels fell after treatment with higher doses of beta-blockers. Additionally, in patients with sST2 concentration above 35 ng/mL, the benefit of high dose beta-blockers was substantially higher than in the group with lower sST2 concentrations [37].

There are limited data about serial sST2 measurements in monitoring treatment effects in patients with CTEPH. So far, well-established markers of heart failure and myocardial damage in clinical practice, such as NT-proBNP and troponin T, were used in monitoring the effects of balloon pulmonary angioplasty in patients with CTEPH [38,39]. However, Kriechbaum et al. revealed increased sST2 levels in 57 patients with inoperable CTEPH compared to healthy controls (53.7 pg/mL (IQR: 45.3–74.1) vs. 48.7 pg/mL (IQR: 35.5–57.0); *p* = 0.02), and sST2 level above 65 ng/mL was strongly associated with advanced hemodynamic impairment prior to BPA treatment in these patients. In their study the median sST2 concentrations decreased to the range of the control group after BPA therapy, but was not associated with the individual degree of therapy response [40]. These observations raise questions about the primary mechanism of sST2 changes in patients with CTEPH. As we know from previous reports, both in cardiac and non-cardiac disease the inflammatory component seems to be the leading driver of sST2 concentration changes [9,41]. Thus, BPA therapy may weaken the ongoing endothelium remodeling and consecutive inflammation by gradually improving hemodynamic parameters, leading to the decrease of sST2 concentration [40]. However, in our study we observed a significant increase in sST2 concentration 24 h and 48 h after BPA treatment in all analyzed procedures, and the reduction of sST2 concentration in relation to that obtained on admission was observed only in the group without postprocedural complications. Our results, including the no increase in NT-proBNP and TnT concentration after procedure, suggest that sST2 may be related to arterial and lung tissue injury. This could be supported by the data obtained by Willems et al. In their study the sST2 concentration significantly increased 24 h after patients underwent peripheral vascular surgery, femoral endarterectomy, or arterial bypass [42]. Additionally, Pascual-Figal et al. reported upregulation of sST2 mRNA in the lung tissue. The authors identified an association between increased alveolus thickness and upregulation of sST2, demonstrating the degree of soluble ST2 production and pulmonary congestion. Thus, sST2 level may be influenced by dynamic contributions of the vascular endothelium and lung tissue. This could be the explanation for the significantly higher sST2 levels in the group that experienced complications in the postprocedural period compared to the group without postprocedural complications. Moreover, one of the previous studies showed that worse hemodynamic parameters could be related with higher complication rate and high pulmonary artery pressure could aggravate the severity of complications [43]. It is worth to emphasize that no relation between BPA complications and acute hemodynamic changes after procedure has been noticed so far. Thus, we hypothesize that the mechanism of sST2 release in patients suffering from complications in the postprocedural period seems to be multifactorial. Potentially it may be associated with both damage to the vascular endothelium, damage to the lung tissue, and excessive RV overload. Our results suggest that sST2 as a comprehensive biomarker may mirror the condition of the heart, lung and pulmonary vascular bed system [44] in patients with CTEPH. Further studies are needed to better define its potential role in monitoring patients undergoing interventional treatment not in only in terms of efficacy, but also in terms of preprocedural risk stratification and postprocedural safety.

This study has several limitations. Firstly, the sample size and procedures analyzed in this study were relatively small. Secondly, despite the observed significant changes in biomarker concentration in the studied population, our results do not provide reliable information on a source of the observed changes in patients with CTEPH. Thirdly, the patients in this study were at different stages of interventional treatment.

## 5. Conclusions

sST2 concentration in CTEPH patients treated with BPA changes significantly in the postprocedural period. sST2 levels are significantly higher in patients who experience complications in the postprocedural period compared to those without complications. Thus, sST2 could be considered as an additional noninvasive tool for monitoring patients undergoing balloon pulmonary angioplasty for complications and risk stratification.

## Figures and Tables

**Table 1:** Baseline characteristics of the general study population.

Variables	All Study Population
Procedures, *n*	57
Age (years)	52 ± 19
WHO FC, *n* (%)	
I	4 (7%)
II	24 (42%)
III	28 (49%)
IV	1(2%)
mRAP (mmHg)	6.9 ± 3.9
mPAP (mmHg)	43.9 ± 10.9
PVR (Wood Unit)	7.6 ± 3.8
CO (L/min)	5.0 ± 1.2
CI (L/min × m^2^)	2.8 ± 0.6
MVsatO2 (%)	67.7 ± 6.3
AOsatO2 (%)	95.6 ± 2.9
sST2 (ng/mL)	26.56 (16.66–40.83)
NT-proBNP (pg/mL)	451 (178–1293)
TnT (ng/mL)	0.009 (0.006–0.017)

Data are presented as *n*; mean and SD or median and IQR. World Health Organization Functional Class—WHO FC. mean right atrial pressure—mRAP, mean pulmonary artery pressure—mPAP, pulmonary capillary wedge pressure—PCWP, cardiac index—CI, pulmonary vascular resistance—PVR, mixed venous oxygen saturation—MVsatO2, aortic oxygen saturation AOsatO2, N-terminal pro brain natriuretic peptide—NT-proBNP, Troponin T—TnT.

**Table 2:** Biomarkers measurements at four time points in the general study population.

Variables	Admission Day	24 h after Procedure	48 h after Procedure	Discharge Day	*p*1 Value	*p*2 Value	*p*3 Value
sST2 (ng/mL)	26.56 (16.66–40.83)	33.31 (20.81–62.56)	27.45 (17.66–54.45)	21.59 (12.50–33.48)	0.048	<0.001	0.028
NT-proBNP (pg/mL)	451 (178–1293)	368 (147–996)	317 (129–851)	240 (132–1077)	0.010	0.006	0.013
TnT (ng/mL)	0.009 (0.006–0.017)	0.01 (0.006–0.019)	0.009 (0.006–0.017)	0.011 (0.006–0.014)	0.44	0.006	0.078

Data are presented as median and IQR. sST2 -soluble ST2, N-terminal pro brain natriuretic peptide -NT-proBNP, Troponin T—TnT; *p*1—admission day vs. discharge day; *p*2—admission day vs. 24 h after procedure; *p*3—admission day vs. 48 h after procedure.

**Table 3:** Hemodynamic parameters in the groups with and without complications in the postprocedural period.

Variables	No Complications in Postprocedural Period	Complications in Postprocedural Period	*p* Value
Procedures, n	42	15	-
mRAP (mmHg)	6.6 ± 3.6	7.6 ± 4.0	0.379
mPAP (mmHg)	41.6 ± 10.1	50.3 ± 10.7	0.009
PVR (Wood Unit)	7.0 ± 3.6	9.5 ± 4.1	0.025
CO (L/min)	5.1 ± 1.3	4.72 ± 1.1	0.285
CI (L/min·m^2^)	2.8 ± 0.6	2.64 ± 0.5	0.320
MVsatO2 (%)	69.1 ± 5.4	63.7 ± 7.1	0.009
AOsatO2 (%)	96.3 ± 2.6	93.7 ± 2.9	0.005

Data are presented as n, mean and SD. World Health Organization Functional Class—WHO FC. mean right atrial pressure—mRAP, mean pulmonary artery pressure—mPAP, pulmonary capillary wedge pressure—PCWP, cardiac index—CI, pulmonary vascular resistance—PVR, mixed venous oxygen saturation—MVsatO2, aortic oxygen saturation—AOsatO2.

**Table 4:** Concentrations of sST2, NT-proBNP, and TnT separately for groups with and without complications in the postprocedural period at four time points.

Variabes		No Complications in Postprocedural Period	Complications in Postprocedural Period	*p*
Admission day	sST2 (ng/mL)	23.25 (15.35–38.69)	39.73 (24.03–47.57)	0.044
	NT-proBNP (pg/mL)	348 (139–1081)	894 (514–2081)	0.003
	TnT (ng/mL)	0.008 (0.00–0.01)	0.014 (0.007–0.04)	0.08
24 h after procedure	sST2 (ng/mL)	26.86 (19.10–40.12)	97.66 (53.07–126.18)	<0.001
	NT-proBNP (pg/mL)	284 (123–530)	996 (445–2466)	0.004
	TnT (ng/mL)	0.009 (0.01–0.014)	0.017 (0.01–0.04)	0.09
48 h after procedure	sST2 (ng/mL)	21.58 (15.73–37.67)	87.35 (52.07–116.41)	<0.001
	NT-proBNP (pg/mL)	185 (114–601)	931 (423–2680)	0.002
	TnT (ng/mL)	0.008 (0.01–0.02)	0.012 (0.01–0.04)	0.08
Discharge day	sST2 (ng/mL)	18.64 (12.50–27.25)	42.97 (28.18–70.20)	<0.001
	NT-proBNP (pg/mL)	195 (103–487)	1077 (348–2099)	0.004
	TnT (ng/mL)	0.01 (0.004–0.014)	0.014 (0.008–0.05)	0.09

Data are presented as median and IQR. Soluble ST2—sST2, N-terminal pro brain natriuretic peptide—NT-proBNP; Troponin T—TnT.

## References

[1] Delcroix M., Noordegraaf A.V., Fadel E., Lang I., Simonneau G., Naeije R. (2012). Vascular and right ventricular remodelling in chronic thromboembolic pulmonary hypertension. Eur. Respir. J..

[2] Simonneau G., Torbicki A., Dorfmüller P., Kim N. (2017). The pathophysiology of chronic thromboembolic pulmonary hypertension. Eur. Respir. Rev..

[3] Galie N., Humbert M., Vachiery J.L., Gibbs S., Lang I., Torbicki A., Simonneau G., Peacock A., Vonk Noordegraaf A., Beghetti M. (2015). 2015 ESC/ERS Guidelines for the diagnosis and treatment of pulmonary hypertension: The Joint Task Force for the Diagnosis and Treatment of Pulmonary Hypertension of the European Society of Cardiology (ESC) and the European Respiratory Society (ERS): Endorsed by: Association for European Paediatric and Congenital Cardiology (AEPC), International Society for Heart and Lung Transplantation (ISHLT). Eur. Respir. J..

[4] Kim N.H., Delcroix M., Jais X., Madani M.M., Matsubara H., Mayer E., Ogo T., Tapson V., Ghofrani H.A., Jenkins D. (2018). Chronic thromboembolic pulmonary hypertension. Eur. Respir. J..

[5] Dieplinger B., Januzzi J.L., Steinmair M., Gabriel C., Poelz W., Haltmayer M., Mueller T. (2009). Analytical and clinical evaluation of a novel high-sensitivity assay for measurement of soluble ST2 in human plasma—The Presage™ ST2 assay. Clin. Chim. Acta.

[6] Dieplinger B., Mueller T. (2015). Soluble ST2 in heart failure. Clin. Chim. Acta.

[7] Coglianese E.E., Larson M.G., Vasan R.S., Ho J.E., Ghorbani A., McCabe E.L., Cheng S., Fradley M.G., Kretschman D., Gao W. (2012). Distribution and Clinical Correlates of the Interleukin Receptor Family Member Soluble ST2 in the Framingham Heart Study. Clin. Chem..

[8] Luk K.S., Ip C., Gong M., Wong S.H., Wu W.K., Dong M., Li G., Chan K.P., Du Y., Liu T. (2017). A meta-analysis of soluble suppression of tumorigenicity 2 (sST2) and clinical outcomes in pulmonary hypertension. J. Geriatr. Cardiol..

[9] Mueller T., Dieplinger B. (2013). The Presage((R)) ST2 Assay: Analytical considerations and clinical applications for a high-sensitivity assay for measurement of soluble ST2. Expert Rev. Mol. Diagn..

[10] Weinberg E.O., Shimpo M., De Keulenaer G.W., MacGillivray C., Tominaga S.-I., Solomon S.D., Rouleau J.-L., Lee R.T. (2002). Expression and regulation of ST2, an interleukin-1 receptor family member, in cardiomyocytes and myocardial infarction. Circulation.

[11] Pei C., Barbour M., Fairlie-Clarke K.J., Allan D., Mu R., Jiang H.-R. (2013). Emerging role of interleukin-33 in autoimmune diseases. Immunology.

[12] Ky B., French B., McCloskey K., Rame J.E., McIntosh E., Shahi P., Dries D.L., Tang W.W., Wu A.H., Fang J.C. (2011). High-Sensitivity ST2 for Prediction of Adverse Outcomes in Chronic Heart Failure. Circ. Hear. Fail..

[13] Pascual-Figal D.A., Manzano-Fernández S., Boronat M., Casas T., Garrido I.P., Bonaque J.C., Pastor-Perez F., Valdés M., Januzzi J.L. (2011). Soluble ST2, high-sensitivity troponin T- and N-terminal pro-B-type natriuretic peptide: Complementary role for risk stratification in acutely decompensated heart failure. Eur. J. Hear. Fail..

[14] Carlomagno G., Messalli G., Melillo R.M., Stanziola A.A., Visciano C., Mercurio V., Imbriaco M., Ghio S., Sofia M., Bonaduce D. (2013). Serum soluble ST2 and interleukin-33 levels in patients with pulmonary arterial hypertension. Int. J. Cardiol..

[15] Daniels L.B., Clopton P., Iqbal N., Tran K., Maisel A.S. (2010). Association of ST2 levels with cardiac structure and function and mortality in outpatients. Am. Hear. J..

[16] Yancy C.W., Jessup M., Bozkurt B., Butler J., Casey D.E., Colvin M.M., Drazner M.H., Filippatos G.S., Fonarow G.C., Givertz M.M. (2017). 2017 ACC/AHA/HFSA Focused Update of the 2013 ACCF/AHA Guideline for the Management of Heart Failure: A Report of the American College of Cardiology/American Heart Association Task Force on Clinical Practice Guidelines and the Heart Failure Society of America. Circulation.

[17] Geenen L.W., Baggen V.J.M., Kauling R.M., Koudstaal T., Boomars K.A., Boersma E., Roos-Hesselink J.W., Bosch A.E.V.D. (2019). The Prognostic Value of Soluble ST2 in Adults with Pulmonary Hypertension. J. Clin. Med..

[18] Zheng Y.-G., Yang T., He J.-G., Chen G., Liu P., Xiong C., Gu Q., Ni X., Zhao Z.-H. (2014). Plasma Soluble ST2 Levels Correlate with Disease Severity and Predict Clinical Worsening in Patients With Pulmonary Arterial Hypertension. Clin. Cardiol..

[19] Kurzyna M., Araszkiewicz A., Błaszczak P., Grabka M., Hawranek M., Kopeć G., Mroczek E., Zembala M., Kamiński K.A., Ochała A. (2015). Summary of recommendations for the haemodynamic and angiographic assessment of the pulmonary circulation. Joint statement of the Polish Cardiac Society’s Working Group on Pulmonary Circulation and Association of Cardiovascular Interventions. Kardiol. Pol..

[20] Araszkiewicz A., DaRocha S., Pietrasik A., Pietura R., Jankiewicz S., Banaszkiewicz M., Sławek-Szmyt S., Biederman A., Mularek-Kubzdela T., Lesiak M. (2019). Balloon pulmonary angioplasty for the treatment of residual or recurrent pulmonary hypertension after pulmonary endarterectomy. Int. J. Cardiol..

[21] DaRocha S., Kurzyna M., Pietura R., Kamiński K.A. (2013). Balloon pulmonary angioplasty for inoperable chronic thromboembolic pulmonary hypertension. Kardiol. Pol..

[22] DaRocha S., Pietura R., Pietrasik A., Norwa J., Dobosiewicz A., Piłka M., Florczyk M., Biederman A., Kamiński K.A., Kurzyna M. (2017). Improvement in Quality of Life and Hemodynamics in Chronic Thromboembolic Pulmonary Hypertension Treated with Balloon Pulmonary Angioplasty. Circ. J..

[23] Kurzyna M., DaRocha S., Pietura R., Pietrasik A., Norwa J., Mańczak R., Wieteska M., Biederman A., Matsubara H., Torbicki A. (2017). Changing the strategy of balloon pulmonary angioplasty resulted in a reduced complication rate in patients with chronic thromboembolic pulmonary hypertension. A single-centre European experience. Kardiol. Pol..

[24] Inami T., Kataoka M., Shimura N., Ishiguro H., Yanagisawa R., Taguchi H., Fukuda K., Yoshino H., Satoh T. (2013). Pulmonary Edema Predictive Scoring Index (PEPSI), a New Index to Predict Risk of Reperfusion Pulmonary Edema and Improvement of Hemodynamics in Percutaneous Transluminal Pulmonary Angioplasty. JACC Cardiovasc. Interv..

[25] Robin X.A., Turck N., Hainard A., Tiberti N., Lisacek F., Sanchez J.-C., Müller M. (2011). pROC: An open-source package for R and S+ to analyze and compare ROC curves. BMC Bioinf..

[26] Pascual-Figal D.A., Januzzi J.L. (2015). The Biology of ST2: The International ST2 Consensus Panel. Am. J. Cardiol..

[27] Lu J., Snider J.V., Grenache D.G. (2010). Establishment of reference intervals for soluble ST2 from a United States population. Clin. Chim. Acta.

[28] Kohli P., Bonaca M.P., Kakkar R., Kudinova A.Y., Scirica B.M., Sabatine M.S., Murphy S.A., Braunwald E., Lee R.T., Morrow D.A. (2012). Role of ST2 in Non–ST-Elevation Acute Coronary Syndrome in the MERLIN-TIMI 36 Trial. Clin. Chem..

[29] Lassus J., Gayat E., Mueller C., Peacock W., Spinar J., Harjola V.-P., Van Kimmenade R., Pathak A., Mueller T., DiSomma S. (2013). Incremental value of biomarkers to clinical variables for mortality prediction in acutely decompensated heart failure: The Multinational Observational Cohort on Acute Heart Failure (MOCA) study. Int. J. Cardiol..

[30] Felker G.M., Fiuzat M., Thompson V., Shaw L.K., Neely M.L., Adams K.F., Whellan D.J., Donahue M.P., Ahmad T., Kitzman D.W. (2013). Soluble ST2 in ambulatory patients with heart failure: Association with functional capacity and long-term outcomes. Circ. Heart Fail..

[31] Banaszkiewicz M., Pietrasik A., DaRocha S., Piłka M., Florczyk M., Dobosiewicz A., Kędzierski P., Pędzich-Placha E., Kochman J., Opolski G. (2020). Soluble ST2 protein as a new biomarker in patientswith precapillary pulmonary hypertension. Arch. Med. Sci..

[32] Haeck M.L., Scherptong R.W., Marsan N.A., Holman E.R., Schalij M.J., Bax J.J., Vliegen H.W., Delgado V. (2012). Prognostic Value of Right Ventricular Longitudinal Peak Systolic Strain in Patients with Pulmonary Hypertension. Circ. Cardiovasc. Imaging.

[33] Fine N.M., Chen L., Bastiansen P.M., Frantz R.P., Pellikka P.A., Oh J.K., Kane G.C. (2013). Outcome Prediction by Quantitative Right Ventricular Function Assessment in 575 Subjects Evaluated for Pulmonary Hypertension. Circ. Cardiovasc. Imaging.

[34] Siennicka A., DaRocha S., Banaszkiewicz M., Kędzierski P., Dobosiewicz A., Błaszczak P., Peregud-Pogorzelska M., Kasprzak J.D., Tomaszewski M., Mroczek E. (2018). Treatment of chronic thromboembolic pulmonary hypertension in a multidisciplinary team. Ther. Adv. Respir. Dis..

[35] Plácido R., Cortez-Dias N., Martins S.R., Almeida A.G., Calisto C., Gonçalves S., Sadoune M., Diogo A.N., Mebazaa A., Pinto F.J. (2017). Prognostic stratification in pulmonary hypertension: A multi-biomarker approach. Rev. Port. Cardiol..

[36] Manzano-Fernández S., Januzzi J., Pastor-Pérez F., Bonaque-González J., Boronat-Garcia M., Pascual-Figal D., Montalban-Larrea S., Navarro-Peñalver M., Andreu-Cayuelas J., Valdés M. (2012). Serial Monitoring of Soluble Interleukin Family Member ST2 in Patients with Acutely Decompensated Heart Failure. Cardiology.

[37] Gaggin H.K., Motiwala S., Bhardwaj A., Parks K.A., Januzzi J.L. (2013). Soluble concentrations of the interleukin receptor family member ST2 and beta-blocker therapy in chronic heart failure. Circ. Heart Fail..

[38] Kriechbaum S.D., Wiedenroth C.B., Keller T., Wolter J.S., Ajnwojner R., Peters K., Haas M.A., Roller F.C., Breithecker A., Rieth A.J. (2018). Dynamics of high-sensitivity cardiac troponin T during therapy with balloon pulmonary angioplasty for chronic thromboembolic pulmonary hypertension. PLoS ONE.

[39] Kriechbaum S.D., Wiedenroth C.B., Wolter J.S., Hütz R., Haas M., Breithecker A., Roller F.C., Kriechbaum S., Guth S., Rolf A. (2018). N-terminal pro–B-type natriuretic peptide for monitoring after balloon pulmonary angioplasty for chronic thromboembolic pulmonary hypertension. J. Heart Lung Transplant..

[40] Kriechbaum S.D., Wiedenroth C.B., Peters K., Barde M.A., Ajnwojner R., Wolter J.-S., Haas M., Roller F.C., Guth S., Rieth A.J. (2020). Galectin-3, GDF-15, and sST2 for the assessment of disease severity and therapy response in patients suffering from inoperable chronic thromboembolic pulmonary hypertension. Biomarkers.

[41] de Boer R.A., Nayor M., de Filippi C.R., Enserro D., Bhambhani V., Kizer J.R., Blaha M.J., Brouwers F.P., Cushman M., Lima J.A. (2018). Association of Cardiovascular Biomarkers with Incident Heart Failure With Preserved and Reduced Ejection Fraction. JAMA Cardiol..

[42] Willems S., Sels J.-W., Flier S., Versteeg D., Buhre W.F., De Kleijn D.P., Hoefer I.E., Pasterkamp G. (2013). Temporal changes of soluble ST2 after cardiovascular interventions. Eur. J. Clin. Investig..

[43] Ejiri K., Ogawa A., Fujii S., Ito H., Matsubara H. (2018). Vascular Injury Is a Major Cause of Lung Injury After Balloon Pulmonary Angioplasty in Patients with Chronic Thromboembolic Pulmonary Hypertension. Circ. Cardiovasc. Interv..

[44] Bayés-Genis A., González A., Lupón J. (2018). ST2 in Heart Failure. Circ. Hear. Fail..

